# scZiva: imputation method for single-cell RNA-seq data with zero-inflated variational autoencoder

**DOI:** 10.1186/s12859-026-06422-2

**Published:** 2026-03-19

**Authors:** Long Tuan Vo, Van Vinh Le, Quoc Toan Ha, Anh Quoc Nguyen

**Affiliations:** Ho Chi Minh City University of Technology and Engineering, Vo Van Ngan Street, Ho Chi Minh City, 700000 Vietnam

**Keywords:** scRNA-seq, Imputation, Missing data, Variational autoencoder

## Abstract

**Background:**

Single-cell RNA sequencing (scRNA-seq) is considered a revolution in gene expression studies and offers significant benefits across various fields of biomedical or clinical applications. However, a high proportion of technical dropouts in scRNA-seq data leads to increased noise and reduces the performance of downstream analyses such as cell clustering, differential expression analysis, and cell trajectory inference. Numerous recent imputation methods utilize deep learning to recover missing gene expression values in scRNA-seq data. Despite the research efforts, existing methods have limitations in capturing local co-expression patterns in scRNA-seq data and handling the uncertainty in distinguishing technical zeros from true biological zeros.

**Results:**

This work proposes a novel imputation method for scRNA-seq data, called scZiva, based on Variational Autoencoder (VAE). It introduces a structured probabilistic framework that jointly models dropout uncertainty and statistically induced local gene dependencies. scZiva also adopts a probability-guided selective imputation mechanism to recover likely technical dropouts while preserving biologically meaningful zeros. The framework is implemented using a Zero-Inflated Negative Binomial (ZINB) likelihood with a convolution-enhanced encoder architecture. Comprehensive experiments conducted on both simulated and real datasets demonstrate the strength of scZiva compared with other baseline methods.

**Conclusion:**

The proposed method demonstrates strong and stable performance in most evaluation settings compared to five baseline methods, particularly in recovering missing gene expression values and supporting downstream analyses. It is a promising approach for analyzing scRNA-seq data.

## Introduction

Gene expression studies have been significantly advanced by single-cell RNA sequencing (scRNA-seq) technologies [1], and have a meaningful impact on biomedical and clinical applications [2]. While traditional bulk RNA-seq technology only provides analysis of gene expression at the population level, scRNA-seq allows for investigating expression profiles at a single-cell resolution. Despite its advantages, scRNA-seq data suffer from some limitations due to their unique characteristics. One of the significant obstacles is a phenomenon called dropout events, which is the state of missing values in data due to technical noises, *i.e.,* amplification bias, low-input cell library, or inefficient mRNA capture. The dropout events can result in poor performance of downstream analysis for scRNA-seq data [3], such as cell clustering, differential expression analysis, and cell trajectory inference. Thus, it poses research challenges for developing effective scRNA-seq imputation methods to recover missing gene expression values.

Most early imputation approaches for scRNA-seq data can be classified into model-based, smoothing-based, and low-ranked matrix-based methods. SAVER [4] and scImpute [5] are typical algorithms of the model-based group, which assume the count of genes in cells follows statistical models. They estimate the parameters of the models using observed data and then infer missing values. While SAVER uses the Poisson-Gamma model and calculates the posterior mean to recover expression values, scImpute is based on a mixture model of Gamma and Normal distributions to represent dropout values and the actual gene expression levels, respectively. Smoothing-based imputation methods, such as MAGIC [6] and AcImpute [7], utilize the similarity between cells when they estimate missing values via neighboring cells. MAGIC firstly constructs an exponential Markov matrix and then achieves an imputed matrix by multiplying the matrix by the original gene expression data. AcImptute constrains smoothing weights across cells for genes that have different expression levels to enhance imputation performance.

Additionally, some low-ranked matrix-based methods, such as CMF-Impute [8], scRMD [9], and netNMF-sc [10], are based on matrix decomposition techniques to map gene expression data into a low-rank representation to support reconstructing missing values in the original matrix. CMF-Impute applies a collaborative matrix decomposition method for two matrices of cell features and gene features in its data recovery task, while scRMD uses a robust matrix decomposition to impute data. Different from the two methods, netNMF-sc additionally uses available gene–gene interactions information to guide its parameter estimation process of the imputation model.

Recent imputation methods for scRNA-seq data primarily utilize the advantages of deep learning techniques. A group of methods, such as sc-fGAIN [11], scMultiGAN [12], Scmaskgan [13], and scGGAN [14], rely on generative adversarial networks (GANs) in their imputation process. In those approaches, two networks of Generator and Discriminator are trained together to help the Generator return a high-quality imputed matrix. sc-fGAIN applies *f*-GAN framework, which utilizes *f*-divergence functions to optimize the imputation model, while both scMultiGAN and Scmakgan transform gene expression matrices into images before performing GAN-based imputation. scGGAN is a graph-based generative adversarial network and incorporates bulk RNA-seq and gene sequencing data for training its model.

Another category of deep learning-based methods for scRNA-seq data imputation, e.g., IGSimpute [15], Bubble [16], DCA [17], and SAE-Impute [18] aim to impute data using autoencoder structures where the decoder is responsible for reconstructing completed data. IGSimpute and Bubble adopt denoising autoencoders, whereas the Bubble model is further constrained by bulk RNA-seq data. While DCA proposes a variant of an autoencoder to denoise scRNA-seq data, supporting the recovery of missing values, SAE-Impute combines autoencoders and subspace regression in its imputation process. Besides, the study of Widad [19] applies a standard Variational Autoencoder to impute scRNA-seq data.

Despite their success, existing deep learning methods still have main limitations. First, most approaches are difficult to explicitly handle the uncertainty in distinguishing technical zeros from true biological zeros. Second, gene expression values are often treated as independent input features, ignoring local co-expression patterns. These limitations are especially evident in current VAE-based frameworks, where reconstruction is typically performed globally without selective treatment of likely dropout events. Furthermore, there is a fundamental trade-off between recovering missing expression signals and preserving true biological silence.

Motivated by these challenges, we propose scZiva, a structured probabilistic imputation framework built on a VAE for scRNA-seq data. The novelty of scZiva lies in a joint probabilistic-structural design with three major characteristics: (i) a ZINB-based likelihood to model overdispersion and zero inflation; (ii) convolutional encoding combined with gene reordering to exploit local co-expression structures; and (iii) a probability-guided selective imputation mechanism that adaptively reconstructs likely technical dropouts while preserving biologically meaningful zeros. Experimental results on both simulated and real datasets show the strength of scZiva compared to baseline methods in various tasks, including the recovery of dropout values, cell clustering, differential expression analysis, and cell trajectory inference.

## Methods

**Fig. 1:**
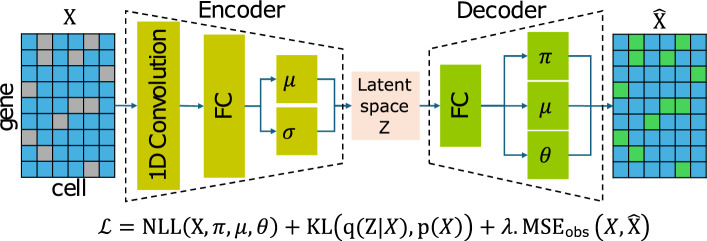
Overall architecture of scZiva. The encoder maps the raw input $\textbf{X}$ to latent parameters $(\mu _Z,\sigma _Z^2)$, and the decoder produces ZINB parameters $(\pi ,\mu ,\theta )$ to reconstruct $\widehat{\textbf{X}}$. Training minimizes a loss combining ZINB negative log-likelihood, KL divergence, and an observed-entry mean squared error term

Given a raw scRNA-seq count matrix $\textbf{X} \in \mathbb {R}^{N \times G}$, where *N* and *G* denote the number of cells and genes, respectively. The objective of scZiva is to recover the expression levels of genes that were not observed due to dropout events. A preprocessing task is applied to discard genes with zero expression across all cells of $\textbf{X}$. The main architecture of scZiva is illustrated in Fig. 1, consisting of an encoder and a decoder. The encoder employs a one-dimensional convolutional layer (Conv1D) to capture those local relationships while the decoder reconstructs the gene expression distribution using the Zero-Inflated Negative Binomial (ZINB [20]) parameterization.

### Encoder: Conv1D for discovering gene relationships

Conv1D layer applies sliding filters along one gene vector dimension to extract local patterns and is widely adopted as a fundamental component in hybrid deep learning architectures [21–23]. Motivated by these successes, we employ Conv1D to explore local gene-gene dependencies that potentially reflect biological patterns. Moreover, to better exploit these local correlations, we first reorder genes using covariance-based hierarchical clustering, ensuring that highly co-expressed genes are positioned adjacently in the input matrix. This preprocessing step introduces structured proximity among statistically related genes, allowing Conv1D to model local co-expression patterns. Noticed that such locality is induced by statistical structure instead of assumed to reflect inherent biological contiguity. Technically, a Conv1D layer with kernel size *k*, stride *s*, and no padding, parameterized by weights $\textbf{w} \in \mathbb {R}^k$ and bias $b \in \mathbb {R}$ shared across all cells, transforms the input as:

1 $$\begin{aligned} \textbf{H}_{i,j} = \sum _{m=0}^{k-1} w_m \, \textbf{X}_{i,\, j s + m} + b, \end{aligned}$$

where $\textbf{H} \in \mathbb {R}^{N \times L}$ is the resulting feature map, *i* indexes the cell ($1 \le i \le N$), *j* indexes the convolutional window, and $L = \left\lfloor \frac{G - k}{s} \right\rfloor + 1$ is the output length along the gene dimension.

Let $\textbf{x}_i \in \mathbb {R}^G$ denote the zero-filled expression profile of cell *i*. The Conv1D output $\textbf{h}_i \in \mathbb {R}^L$ is passed through a fully connected layer to produce a hidden representation, which is then linearly projected into the mean vector $\boldsymbol{\mu }_i \in \mathbb {R}^{d_z}$ and log-variance vector $\boldsymbol{\log \sigma }^2_i \in \mathbb {R}^{d_z}$ of the approximate posterior:

2 $$\begin{aligned} q(\textbf{Z}_i \mid \textbf{X}_i) = \mathcal {N}\!\left( \boldsymbol{\mu }_i, \textrm{diag}\!\big (\exp (\boldsymbol{\log \sigma }^2_i)\big )\right) . \end{aligned}$$

Sampling from this posterior is performed via the reparameterization trick:

3 $$\begin{aligned} \textbf{Z}_i = \boldsymbol{\mu }_i + \boldsymbol{\sigma }_i \odot \boldsymbol{\epsilon }, \quad \boldsymbol{\epsilon } \sim \mathcal {N}(\textbf{0}, \textbf{I}). \end{aligned}$$

### Decoder: ZINB parameterization for gene expression reconstruction

Given a latent representation $\textbf{Z}_i \in \mathbb {R}^{d_z}$ from the encoder, the decoder maps it back to the gene expression space by predicting the parameters of a ZINB distribution for each gene [20]. Specifically, the decoder projects $\textbf{Z}_i$ through a fully connected layer with a rectified linear activation, producing a hidden representation. This is followed by another linear transformation whose output dimension is 3*G*, yielding a vector $\textbf{o}_i \in \mathbb {R}^{3G}.$ We then partition $\textbf{o}_i$ into three *G*-dimensional components corresponding to dropout probability, mean, and dispersion:

$$ \textbf{o}_i = \big [\,\textbf{p}^{(\pi )}_i \;\Vert \; \textbf{p}^{(\mu )}_i \;\Vert \; \textbf{p}^{(\theta )}_i \,\big ], $$

where $\Vert \cdot \Vert$ denotes concatenation. To ensure valid parameter domains, element-wise nonlinearities are applied:

4 $$\begin{aligned} \boldsymbol{\pi }_i&= \sigma \!\left( \textbf{p}^{(\pi )}_i\right) , (\boldsymbol{\mu }_i,\boldsymbol{\theta }_i)= \textrm{softplus}\!\left( \textbf{p}^{(\mu )}_i,\textbf{p}^{(\theta )}_i\right) , \end{aligned}$$

with $\sigma (\cdot )$ the logistic sigmoid mapping to (0, 1) and $\textrm{softplus}(\cdot )=\log (1+e^{(\cdot )})$ ensuring strictly positive outputs.

Thus, for each cell *i* and gene *j*, the decoder outputs a triplet $(\pi _{ij}, \mu _{ij}, \theta _{ij})$ parameterizing the ZINB distribution:

5 $$\begin{aligned} X_{ij} \sim \textrm{ZINB}\left( \pi _{ij}, \mu _{ij}, \theta _{ij}\right) , \end{aligned}$$

where $\pi _{ij}$ models the dropout probability, $\mu _{ij}$ represents the mean expression, and $\theta _{ij}$ controls the inverse dispersion. Details about formula (5) are provided in the Supplementary Section 1. During training, these three parameters are optimized jointly with the encoder via a composite loss that includes the ZINB negative log-likelihood, Kullback–Leibler divergence in the latent space, and a small mean squared error term for reconstruction.

### Training phase

The training objective of scZiva extends the standard VAE framework by combining probabilistic modeling of count data with an auxiliary reconstruction criterion. Formally, the loss function is defined as:

6 $$\begin{aligned} \mathcal {L} = \mathcal {L}_{\text {ZINB}} + \mathcal {L}_{\text {KL}} + \lambda \cdot \mathcal {L}_{\text {MSE}}, \end{aligned}$$

where each term is described below.

*ZINB negative log-likelihood* The primary objective here is to minimize the negative log-likelihood of the observed counts under the ZINB distribution parameterized by $(\pi _{ij}, \mu _{ij}, \theta _{ij})$. Formally, for each entry $x_{ij}$, the log-likelihood is defined as:

7 $$\begin{aligned} \ell (X_{ij}) = {\left\{ \begin{array}{ll} \log \!\Big ( \pi _{ij} + (1-\pi _{ij}) \cdot \text {NB}(0;\mu _{ij},\theta _{ij}) \Big ), & X_{ij}=0, \\ \log (1-\pi _{ij}) + \log \text {NB}(X_{ij};\mu _{ij},\theta _{ij}), & X_{ij}>0, \end{array}\right. } \end{aligned}$$

where $\text {NB}(X;\mu ,\theta )$ denotes the probability mass function of the Negative Binomial distribution. The ZINB loss is then the negative sum of log-likelihoods across all cells and genes:

8 $$\begin{aligned} \mathcal {L}_{\text {ZINB}} = - \sum _{i=1}^N \sum _{j=1}^G \ell (X_{ij}). \end{aligned}$$

This term ensures the model can learn to distinguish biological zeros from technical dropouts (via $\pi _{ij}$), as well as the overdispersed distribution of expression counts (via $\mu _{ij}$ and $\theta _{ij}$).

*Kullback–Leibler divergence* $\mathcal {L}_{\text {KL}}$ is the Kullback–Leibler divergence between the approximate posterior $q(\textbf{Z}_i \mid \textbf{X}_i)$ and the standard Gaussian prior $\mathcal {N}(\textbf{0}, \textbf{I})$. This term makes the latent space smoother and prevents overfitting:

9 $$\begin{aligned} \mathcal {L}_{\text {KL}}=\frac{1}{2}\sum _{k=1}^{d_z}(\mu _{ik}^2+\sigma _{ik}^2-\log \sigma _{ik}^2-1). \end{aligned}$$

*Auxiliary MSE loss* In addition to the probabilistic objective, we introduce a mean squared error (MSE) term that directly compares the expected value of the ZINB reconstruction with the observed counts on non-dropout event entries:

10 $$\begin{aligned} \mathcal {L}_{\text {MSE}} = \sum _{(i,j) \in \Omega } \left[ (1 - \pi _{ij}) \cdot \mu _{ij} - x_{ij} \right] ^2, \end{aligned}$$

where $\Omega$ denotes the set of observed (non-dropout) indices. This auxiliary loss provides an explicit reconstruction signal that complements the statistical likelihood, encouraging the latent representation to retain richer semantics beyond what is strictly required by maximum likelihood. Importantly, this auxiliary term does not replace the ZINB likelihood as the primary training objective, but serves as a complementary regularizer applied only to observed (non-dropout) entries. Since the MSE is restricted to $\Omega$ and weighted adaptively, it does not enforce global smoothing across all zero entries. Such auxiliary objectives have been shown to improve representation quality and generalization [24, 25], similar to the role of dual self-supervised objectives in approaches such as MVEB [26].

*Adaptive weighting* Instead of fixing the coefficient $\lambda$ manually, scZiva introduces a learnable weight $\lambda$ that adaptively balances the auxiliary MSE loss during training. This weight is jointly optimized with the model parameters and regularized by a small quadratic penalty $\lambda _{\text {reg}}(\lambda - 1)^2$ to prevent divergence from its nominal value.

### Imputation strategy

After training, scZiva leverages the learned parameters of the ZINB decoder to recover missing or dropout values in the expression matrix. Let $\boldsymbol{\pi }$ and $\boldsymbol{\mu }$ denote the estimated dropout probability and mean expression matrices, respectively. Here, $\pi _{ij}$ represents the posterior probability under the model that entry (*i*, *j*) arises from technical dropout. For each cell *i* and gene *j*, a zero entry in the input matrix is considered a potential dropout. Imputation is then performed selectively according to a threshold $\tau$ on the dropout probability:

11 $$\begin{aligned} \widehat{X}_{ij} = {\left\{ \begin{array}{ll} (1 - \pi _{ij}) \cdot \mu _{ij}, & \text {if } X_{ij}=0 \ \text {and}\ \pi _{ij}>\tau , \\ X_{ij}, & \text {otherwise}. \end{array}\right. } \end{aligned}$$

The threshold $\tau$ acts as a decision boundary that controls how many zero entries are imputed; in other words, it preserves statistically meaningful zero expressions. By setting $\tau$ to a very small value, scZiva restricts the zero-imputations that are assigned by the ZINB decoder. Notably, this mechanism does not assert biological truth for any specific zero entry; instead, it provides a probabilistically guided selection strategy under the model assumptions. Additionally, the scZiva algorithm is presented in detail in Supplementary Algorithm 1.

### Evaluation metrics

This work utilizes three commonly used matrices of Root Mean Squared Error (RMSE), Mean Absolute Error (MAE), and Pearson Correlation Coefficient (PCC) for evaluating the imputation quality of scZiva and baseline methods. While RMSE measures the average magnitude of the squared differences between predicted values and true counts, MAE calculates the average absolute difference between the predicted and true ones. Otherwise, PCC assesses the linear correlation between predicted and true values. More specifically, given the gene expression matrix $\textbf{X} \in \mathbb {R}^{N \times G}$, and an imputed matrix $\widehat{\textbf{X}} \in \mathbb {R}^{N \times G}$ with *N* cells and *G* genes, the three metrics can be calculated as the following formulations [27, 28].

12 $$\begin{aligned} \text {RMSE}&= \sqrt{\frac{1}{N \cdot G} \sum _{i=1}^{N} \sum _{j=1}^{G} \big ( X_{ij} - \widehat{X}_{ij} \big )^2}, \end{aligned}$$

13 $$\begin{aligned} \text {MAE}&= \frac{1}{N \cdot G} \sum _{i=1}^{N} \sum _{j=1}^{G} \big | X_{ij} - \widehat{X}_{ij} \big |, \end{aligned}$$

14 $$\begin{aligned} \text {PCC}&= \frac{ \displaystyle \sum _{i=1}^{N} \sum _{j=1}^{G} \big ( X_{ij} - \overline{X} \big ) \big ( \widehat{X}_{ij} - \overline{\widehat{X}} \big ) }{ \displaystyle \sqrt{ \sum _{i=1}^{N} \sum _{j=1}^{G} \big ( X_{ij} - \overline{X} \big )^2 } \sqrt{ \sum _{i=1}^{N} \sum _{j=1}^{G} \big ( \widehat{X}_{ij} - \overline{\widehat{X}} \big )^2 } }. \end{aligned}$$

In downstream analyses, this work uses Adjusted Rand Index (ARI) [29] and Normalized Mutual Information (NMI) [30] to evaluate the performance of clustering methods on raw and imputed gene expression data. In addition, Kendall’s rank correlation score [31] is used to assess the cell trajectory inference, while the overlap proportion of genes is utilized to evaluate the results of differential expression analysis.

### Benchmark settings

#### Baseline methods and hyperparameter settings

The proposed method is compared to five baseline methods, including AcImpute, SAVER, scRMD, CL-Impute, and SAE-Impute. These methods were selected because they are widely recognized state-of-the-art imputation tools across different methodological categories. In detail, AcImpute is a smoothing-based algorithm, while SAVER is a model-based technique, and scRMD is a low-rank matrix-based method. The remaining approaches, CL-Impute and SAE-Impute, are deep learning-based algorithms.

In the scZiva model, Conv1D is adopted with the kernel size set to $k=64$, the hidden and latent dimensions are fixed to 128 and 64, respectively. The model is trained for 200 epochs using the Adam optimizer with a learning rate of $10^{-3}$. An auxiliary reconstruction loss is incorporated, whose weight is learned during training and constrained within the range [0.5, 1.5], with a regularization coefficient $\lambda _{\text {red}}=10^{-3}$. For imputation, the dropout probability threshold is set to $\tau =10^{-3}$ to selectively recover dropout events. An evaluation of $\tau$ is presented in Supplementary Section 10. All hyperparameters are kept identical across datasets.

Additionally, scZiva and all baseline imputation methods are run five times independently with different random seeds set to 1, 12, 123, 1234, and 12345, respectively, to ensure the reliability of experimental results. The mean values of RMSE, MAE, and PCC from the five runs are reported as the imputation performance. In the case of generating imputed data for downstream analysis, the random seed is set to 1 for all cases.

#### Datasets and data processing

It is a fact that the ground truth of dropout events in real datasets is not available. Therefore, simulated datasets generated by Splatter tool [32] are employed to provide reliable reference values. In addition, real scRNA-seq datasets are used to assess the effectiveness of imputation through downstream analyses. In particular, eight simulated datasets are generated based on Tung dataset [33] with missing rates ranging from 20% to 90%. Tung dataset is a real dataset, provided by default with Splatter tool, has 564 cells and 13.106 genes. Furthermore, 14 real datasets (12 single-cell RNA datasets and 2 bulk RNA datasets), presented in detail in Supplementary Section 3, are used to evaluate the support of scZiva and baseline methods for downstream analysis tasks.

For a fair comparison, all baseline methods are applied to the same gene filtering step, where all genes not expressed in any cell are discarded. Besides, the gene or cell filtering (if any) in the baseline methods is skipped to ensure the dimensions of the imputed data generated by the different methods are the same. Furthermore, we also apply the tasks of log2-transform and data normalization to all imputation methods but respect their original procedure (same as in [34]). In particular, if these two tasks are included in the methods, they are performed without modification. In the cases where a method does not perform one or both of these tasks throughout its algorithms, the remaining unperformed tasks are applied to the imputed datasets.

## Results

### scZiva effectively recovers missing values

Our method and five baseline methods are tested on eight datasets corresponding to different dropout rates from 20% to 90%, where the ground truths are known for assessment. Table 1 presents MAE scores of the imputation methods on the datasets. It can be seen from the table that scZiva achieves the best MAE results among the methods for all cases of dropout rates. Particularly, in the case of 70% dropout rates, the proposed method gets better MAE scores from 0.02 to 1.66 compared to the baseline methods. In addition, RMSE values of the imputation methods for the datasets are presented in Supplementary Table S2. scZiva achieves the lowest RMSE values in 6 out of 8 cases (dropout rates from 30% to 80%), and ranks second in the remaining cases (dropout rates 20% and 90%).

**Table 1:** MAE values of scZiva and five baseline methods under different dropout rates

	Dropout rate							
Method	20%	30%	40%	50%	60%	70%	80%	90%
CL-Impute	0.0201	0.0322	0.0472	0.0645	0.0807	0.1012	0.1255	0.1727
scRMD	0.3260	0.4077	0.5172	0.6218	0.7938	1.1246	1.6408	2.6686
SAE-Impute	0.2653	0.2757	0.2868	0.2989	0.3110	0.3241	0.3342	0.3356
AcImpute	0.8853	1.0848	1.2202	1.4572	1.5003	1.7294	1.8394	1.8823
SAVER	0.0030	0.0071	0.0162	0.0329	0.0551	0.0914	0.1387	0.2145
scZiva	**0**.**0029**	**0**.**0062**	**0**.**0126**	**0**.**0245**	**0**.**0409**	**0**.**0706**	**0**.**1099**	**0**.**1809**

The best results are highlighted in bold

**Fig. 2:**
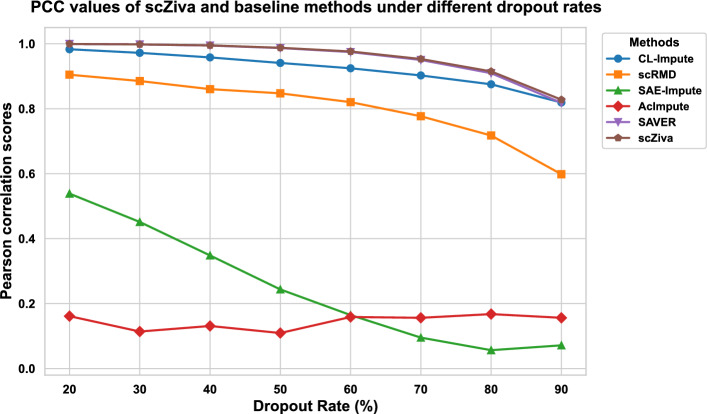
PCC values of scZiva and five baseline methods under different dropout rates

Figure 2 shows that the proposed method has the highest PCC values for all dropout rates. The performance of all methods decreases when dropout rates increase. It can be understood that higher dropout rates make the imputation more difficult. SAE-Impute and AcImpute have the lowest performance among the methods. While the performance of SAE-Impute declines significantly as the dropout rate increases, AcImpute is less affected by the increase in dropout rates, but it has the lowest PCC values for most cases.

### scZiva improves cell type clustering

The clustering of cells is a considerable downstream task in scRNA-seq analysis, which aims to classify cells into groups of similar gene expression levels [35]. This experiment assesses the ability to support cell clustering of the imputation methods. scZiva and five baseline methods are firstly used to perform 10 real datasets (presented in Supplementary Table S1) to generate imputed expression matrices. The imputed matrices are then preprocessed by PCA [36] and clustered by k-means algorithm [37]. Besides, the same clustering task is also applied for the raw datasets, which are not imputed by any imputation methods.

**Fig. 3:**
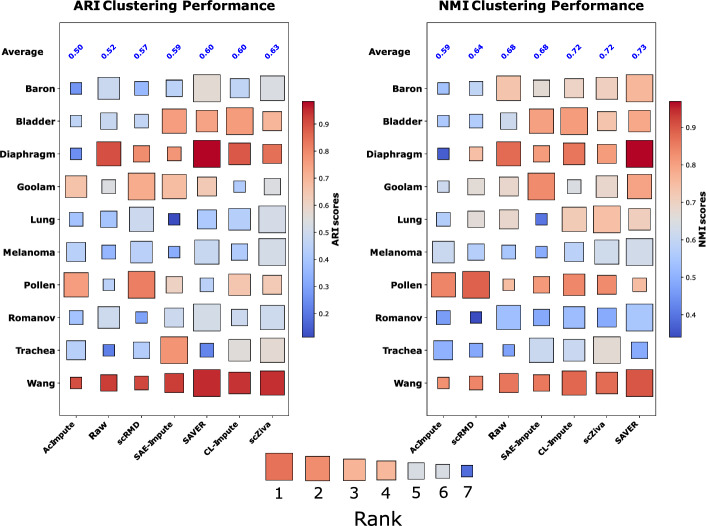
The performance of different imputation methods in supporting the cell clustering

Figure 3, Supplementary Table S3, and Table S4 present ARI and NMI scores of k-means on the imputed and raw datasets. It can be observed from the heatmaps that scZiva, SAVER, SAE-Impute, and CL-Impute are the four methods that are able to give better both average ARI and NMI scores than the case of the raw dataset. SAVER is the best algorithm among the methods, while scZiva is in second place with 6 of the 10 ARI scores and 3 of the 10 NMI scores ranking within the top two. Moreover, the proposed method achieves the highest average ARI and the second-highest average NMI among the methods.

### scZiva supports the differential expression analysis

As one of the common downstream analyses of scRNA-seq data, the analysis of differential gene expression provides meaningful information to deeply understand diseases or distinguish cell types. The task aims to reveal the differentially expressed genes (DEGs) that show different activity levels between two or more groups of cells or tissues. In this work, we evaluate the impact of scZiva and the five baseline imputation methods on identifying differentially expressed genes. Following the process in previous studies [34], the Wilcoxon rank-sum test algorithm [38] is used to calculate DEGs for raw and imputed single-cell datasets. In addition, DEGs identified from bulk RNA-seq samples by the *limma* package [39] are treated as "gold standard" to assess results on single-cell samples with adjusted P-value set to smaller than 0.05.

This experiment is performed on a real dataset available from the GEO database under the Series accession number GSE75748 [40]. The dataset, presented in Supplementary Section 3.2, consists of 1018 cells belonging to 7 cell types (H1, H9, EC, NPC, DEC, HFF, and TB), and has both single-cell and bulk samples. scZiva and five baseline methods are first used to generate six imputed scRNA-seq datasets. The DEGs in a raw scRNA-seq dataset, six imputed datasets, and a bulk sample are then calculated by the corresponding algorithms. We select the top 30 DEGs from scRNA-seq and bulk data to compare the overlapping levels.

**Fig. 4:**
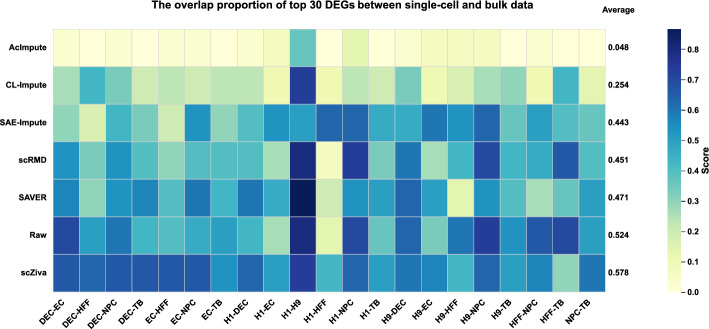
The performance of scZiva and five baseline methods in supporting the identification of differentially expressed genes in GSE75748 dataset. Heatmap showing the value of overlap proportion for the top 30 DEGs between single-cell and bulk data. Each row shows imputation methods, while each column presents pairs of cell types

The top 30 DEGs overlap proportion of all pairs of cell types from the results on single-cell and bulk datasets is presented in Fig. 4. It can be observed that scZiva achieves the best results for 11/21 pairs. Separately compared with the case of raw dataset (without imputation), scZiva gets 13/21 better cases while SAE-Impute has 2/21 higher cases. SAVER and scRMD have only one pair, which has better overlap values compared with the case of raw data. On average, our method gets the highest overlap proportion, and improves 5.4% overlap values compared with the case of not using imputation. Additional experiments for the cases of different top DEGs, presented in Supplementary Figure S1, also illustrate the strength of the proposed methods for the differential expression analysis.

### scZiva significantly enhances the cell trajectory inference

The cell trajectory inference task allows us to understand the dynamic development of cells, as well as aids in identifying new cell subpopulations [41]. In order to assess the support of scZiva on the task, this work performs experiments on the time-course scRNA-seq dataset (GSE75748), which differentiates from human embryonic stem cells to definitive endoderm in a time-course manner at 0, 12, 24, 36, 72, and 96 h. We use Monocle2 package [42] to calculate and visualize the cell trajectory and infer pseudo-time for raw and imputed datasets. The Kendall’s rank correlation metric is used to assess the rank-based associations between predicted pseudo-time and true-time labels.

**Fig. 5:**
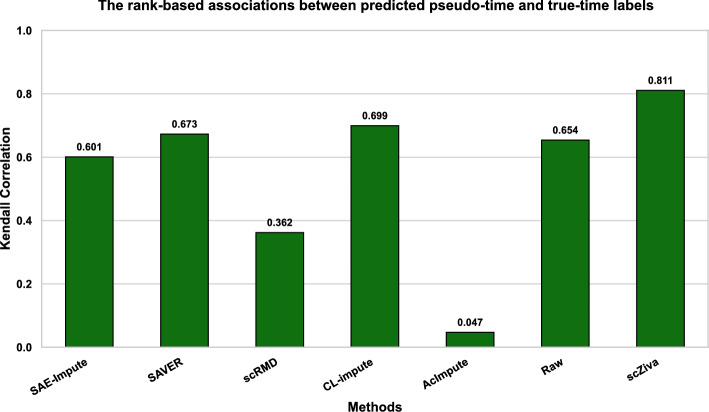
The performance of imputation methods in the support of cell trajectory inference, and the case of raw GSE75748 dataset

**Fig. 6:**
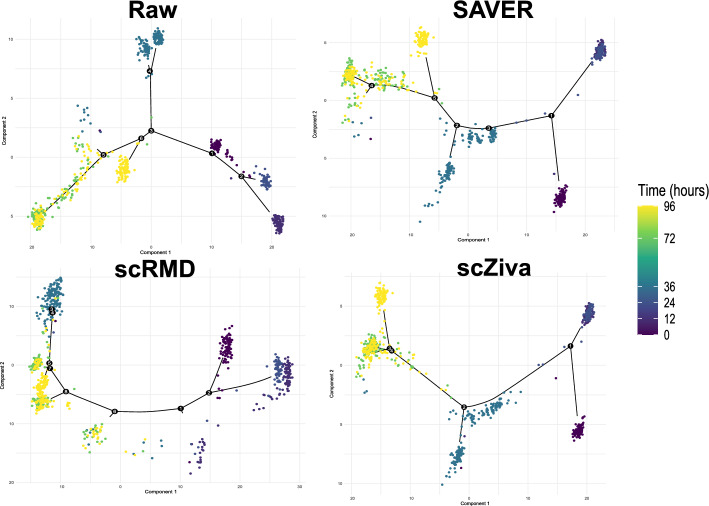
The visualization of trajectory by time with the support of imputation methods (SAVER, scRMD, and scZiva), and the case of raw GSE75748 dataset

The bar chart in Fig. 5 presents Kendall correlation scores of the cell trajectory supported by the methods. It can be seen that our method gets the highest scores and is one of three methods (others are CL-Impute, and SAVER) that are able to improve the cell trajectory inference ability compared with the case of not using imputation. scRMD, AcImpute, and SAE-Impute reduce the cell trajectory performance compared with the case of the raw dataset. The visualization of the trajectory results in Fig. 6 and Supplementary Figure S14 illustrates that the case of scZiva produces a predicted cell trajectory with clearer pseudotime ordering compared to the other cases.

### Ablation study

**Fig. 7:**
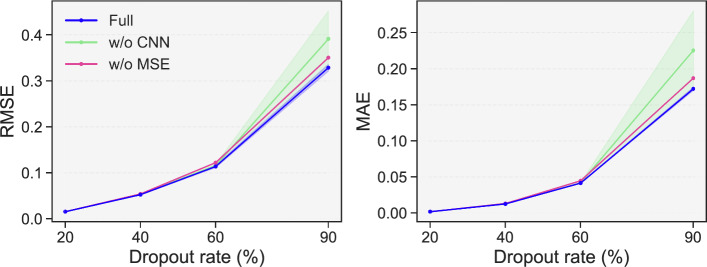
The performance of the full scZiva model and its ablated variants under different dropout rates of Tung dataset. The full model generally achieves the best reconstruction accuracy

To validate the contribution of each component in scZiva, this work conducts an ablation study by removing key modules from the full model. Specifically, scZiva full model is compared with two variants: w/o CNN (removing the Conv1D module), and w/o MSE (removing the auxiliary MSE). All variants and scZiva are trained under identical settings on 20 random seeds across four dropout rates of Tung dataset. The average and standard deviation of RMSE and MAE returned by the methods are presented in Fig. 7.

The line charts show that scZiva consistently achieves the lowest RMSE and MAE across all dropout rates. At low dropout levels (20% to 40%), the performance advantage of the full model over its variants is less pronounced. However, this advantage becomes increasingly evident as the dropout rate increases. Removing the CNN-based module leads to higher errors and larger performance fluctuations, indicating that modeling local gene dependencies provides practical benefits for imputation. Similarly, removing the auxiliary MSE loss results in progressively larger reconstruction errors. We also show the statistical analysis using the Friedman test, followed by pairwise one-sided Wilcoxon signed-rank tests (with Bonferroni correction). The tests confirm that these differences are significant ($p < 0.05$) for all metrics, as shown in Table 2. These results demonstrate that each component of scZiva plays an important role in accurate imputation.

**Table 2:** Statistical significance testing between the full scZiva model and its ablated variants. Overall differences are assessed using the Friedman test, followed by one-sided Wilcoxon signed-rank tests with Bonferroni correction. Effect size is reported as *r*

Metric	Comparison	$p_{\text {raw}}$	$p_{\text {adj}}$	*r*
RMSE	Friedman	$1.25\times 10^{-12}$	–	–
	Full vs. w/o CNN	$5.41\times 10^{-3}$	$1.62\times 10^{-2}$	0.62
	Full vs. w/o Aux	$9.54\times 10^{-7}$	$2.86\times 10^{-6}$	1.10
MAE	Friedman	$2.31\times 10^{-12}$	–	–
	Full vs. w/o CNN	$1.50\times 10^{-2}$	$4.50\times 10^{-2}$	0.54
	Full vs. w/o Aux	$9.54\times 10^{-7}$	$2.86\times 10^{-6}$	1.10

## Discussion

Recovering gene expression values provides reliable biological information and supports the downstream analyses. The proposed method shows the strength in reconstructing missing values through different levels of dropout rates. This imputation is meaningful as it demonstrates the ability to improve the quality of downstream analytical tasks. In particular, compared with the case of performing on raw datasets, analyzing results on imputed datasets by scZiva return better performance for all cases of cell clustering, identifying the differentially expressed genes, and inferring cell trajectories. Compared with baseline methods, the proposed method shows competitive performance when being one of the two best methods for supporting the cell clustering task, and achieving the highest performance in the two remaining downstream analyses.

It is worth noting that the simulated datasets generated by Splatter are based on NB/ZINB-like assumptions, which are partially aligned with the decoder of scZiva. While such alignment helps us to control the quantitative evaluation under known ground truth, it may favor methods with a similar distribution. Therefore, the real scRNA-seq datasets analyzed in this study serve as a more comprehensive validation of the practical effectiveness of the proposed method.

The experiments for the cell trajectory inference illustrate that current imputation methods provide limited support to improve DEGs identification. These results are consistent with the observations reported in the benchmarking study [34]. Therefore, the improvement achieved by scZiva compared to the case of not using imputation is notable. However, further studies are needed to explore the reasons behind this limitation and to enhance the DEGs identification performance.

This work also shows that exploiting local gene-level features is beneficial in the analysis of scRNA-seq data. The integration of Conv1D layers into the VAE encoder significantly enhances the imputation quality. Moreover, although computational efficiency is not the primary concern of this study, experimental results shown in Supplementary Table S6 indicate that the computational aspect is also a strength of the proposed method.

Considering the effect of scZiva on widely studied genes, we further conducted an experimental analysis to examine how scZiva handles zero values in known housekeeping genes (*HPRT1, SDHA*) and cell type-specific marker genes (*CXCR4, CD34, SOX2*). *SOX2* is also a transcription factor. Supplementary Figures S3 to S13 in Supplementary Section 7 present that the proposed method imputes zero values without altering the biological characteristics of the genes. While the constitutive expression of the observed housekeeping genes is preserved, the analyzed marker genes remain highly enriched in their respective cell types. Thus, the biological meaning of gene expression is preserved after imputation. Furthermore, the expression level of the genes in the imputed data appears more consistent with bulk data compared to the raw data. However, further investigation into the impact of imputation on lowly expressed yet biologically important genes, such as transcription factors, may provide valuable insights for the improvement of imputation approaches.

While scZiva demonstrates strong performance across multiple benchmarks, it should be viewed as a methodological improvement within the current landscape of imputation approaches to scRNA-seq data. The complex biological and technical variability of single-cell transcriptomics remains difficult to fully capture within a single modeling framework. Future developments may integrate additional biological or multi-modal information to further enhance interpretability and robustness.

## Conclusion

Dropout events in scRNA-seq caused by technical noise require effective methods to recover true expression values for enhancing the quality of downstream data analysis tasks. This study proposes an accurate scRNA-seq imputation method using a zero-inflated variational autoencoder. The main contributions of the proposed method includes leveraging ZINB distribution augmented with an auxiliary MSE term, as well as exploiting local gene-gene relationships to better preserve meaningful co-expression patterns using Conv1D layers. Furthermore, a probability-driven imputation strategy is also applied to enable an adaptive reconstruction of missing values in scRNA-seq data. The proposed method demonstrates competitive and robust performance compared to five baselines in recovering missing gene expression values and downstream analysis tasks.

## Additional file

Supplementary file 1 (pdf 2009 KB)

## Data Availability

All datasets used in this work can be directly downloaded from https://zenodo.org/records/18263992. The details and original references of the datasets also presented in Supplementary Data file. The source code of the proposed method are published in github page https://github.com/tuanlvo293/scZiva.
