# Supplementary material for: scZiva: imputation method for single-cell RNA-seq data with zero-inflated variational autoencoder
Source: BMC Bioinformatics. 2026 Mar 19;27:92. doi: 10.1186/s12859-026-06422-2 (PMC13122936; doi:10.1186/s12859-026-06422-2)
Supplement: Supplementary file 1 — (pdf 2009 KB) [file 12859_2026_6422_MOESM1_ESM.pdf]

Supplementary Data

scZiva: Imputation method for single cell RNA-seq data with Zero-Inflated Variational Autoencoder

Contents

1. **Zero-Inflated Negative Binomial (ZINB)** 2
2. **Algorithm of scZiva** 2
3. **The details of real datasets** 3
4. **The RMSE values of imputation methods** 4
5. **The details of clustering results** 4
6. **Average overlap proportion over various top DEGs** 5
7. **The expression levels of housekeeping genes and marker genes** 6
8. **Cell trajectory inference of CL-Impute, SAE-Impute and AcImpute** 12
9. **Computational performance** 13
10. **Sensitivity analysis of the threshold τ** 14

1. Zero-Inflated Negative Binomial (ZINB)

scRNA-seq data is characterized by a high frequency of zero counts, arising from both biological silence and technical dropouts. Additionally, the count distributions are often overdispersed, where the variance exceeds the mean, rendering the Poisson model inadequate. To account for these properties, we model each gene expression count using a ZINB distribution.

Let $\pi \in (0; 1)$ denote the zero-inflation probability, $\mu > 0$ the mean of the count distribution, and $\theta > 0$ the inverse dispersion parameter. The probability mass function of ZINB is given by:

$$NB(x; \mu; \theta) = \binom{x + \theta - 1}{x} \left(\frac{\theta}{\theta + \mu}\right)^{\theta} \left(\frac{\mu}{\mu + \theta}\right)^{x}$$

This formulation enables the model to distinguish between "true" zeros and those arising from dropout effects, thereby improving the expressiveness and accuracy of downstream imputation.

2. Algorithm of scZiva

Algorithm 1. scZiva	
**Input:**	Expression matrix X, number of epochs *epochs*
**Output:**	Imputed expression matrix $\hat{X}$
**Procedure:**	$X \leftarrow reorder(X)$ based on gene covariance<br>**for** each epoch in epochs **do**:<br>$Z \leftarrow Encoder(X)$: Latent representation via reparameterization<br>$(\pi, \mu, \theta) \leftarrow Decoder(Z)$: ZINB distribution parameters<br>Loss function: $\mathcal{L} = \mathcal{L}_{ZINB} + \mathcal{L}_{KL} + \lambda . \mathcal{L}_{MSE}$<br>Update parameters using Adam optimizer<br>**end for**<br>Expected expression: $\hat{X} = (1 - \pi) \odot \mu$<br>Binary mask: $M = \mathbb{I}(X = 0) \odot \mathbb{I}(\pi > \tau)$<br>Update matrix: $\hat{X} \leftarrow X + (M \odot \hat{X})$<br>**return** restore_order($\hat{X}$)

3. The details of real datasets

3.1. Datasets used in Clustering tasks

No.	Datasets	Platform	Number of cells	Number of genes	Number of clusters	Source
1	Baron Human	inDrop	8569	20125	14	[1]
2	Bladder	10x	2500	23341	4	[2]
3	Diaphragm	Smart-seq2	870	23341	5	[2]
4	Goolam	Smart-Seq2	124	41480	5	[3]
5	Lung	Smart-Seq2	1676	23341	11	[2]
6	Melanoma	Smart-seq2	4513	23684	9	[4]
7	Pollen	unknown	301	21721	11	[5]
8	Romanov	unknown	2881	24341	7	[6]
9	Trachea	Smart-seq2	1350	23341	4	[2]
10	Wang	10x	9519	14561	2	[7]

***Table S1**. 10 real scRNA-seq datasets.*

3.2. Datasets used in Differential Expression Analysis and Cell trajectory inference

- GSE75748 single-cell: Contains 1018 cells belonging 7 cell types: neuronal progenitor cell (NPC), definitive endoderm cell (DEC), endothelial cell (EC), trophoblast-like cell (TB), human foreskin fibroblasts (HFF), H1 human embryonic stem cells (H1), H9 human embryonic stem cells (H9).

- GSE75748 bulk: Used to assess the DEGs identification performance.

- GSE75748 single-cell time course: The dataset describes the time-course differentiation from human embryonic stem cells to definitive endoderm at 0, 12, 24, 36, 72, and 96 hours.

- GSE75748 bulk time course: Used to assess the cell trajectory inference performance.

The datasets can be downloaded directly from the study [8] or the source:

https://www.ncbi.nlm.nih.gov/geo/query/acc.cgi?acc=GSE75748

4. The RMSE values of imputation methods

Methods	Missing rate (20%)	Missing rate (30%)	Missing rate (40%)	Missing rate (50%)	Missing rate (60%)	Missing rate (70%)	Missing rate (80%)	Missing rate (90%)
**CL-Impute**	0.0937	0.1217	0.1508	0.1801	0.2043	0.2319	0.2627	**0.3249**
**scRMD**	0.9187	0.9961	1.0924	1.1624	1.3425	1.7054	2.2404	3.2239
**SAE-Impute**	0.4701	0.4751	0.4820	0.4909	0.4998	0.5126	0.5286	0.5522
**AcImpute**	2.0098	2.1430	2.1485	2.3143	2.2419	2.3927	2.4211	2.3785
**SAVER**	**0.0152**	0.0315	0.0537	0.0863	0.1232	0.1784	0.2480	0.3620
**scZiva**	0.0161	**0.0306**	**0.0509**	**0.0814**	**0.1161**	**0.1696**	**0.2347**	0.3434

Table S2. *RMSE values of scZiva and five baseline method. Bold values indicate the best results.*

5. The details of clustering results

ARI	AcImpute	Raw	scRMD	SAE-Impute	SAVER	CL-Impute	scZiva
Baron	0.2682	0.4889	0.3586	0.4497	**0.5711**	0.4613	<u>0.5355</u>
Bladder	0.4611	0.4820	0.4697	<u>0.7562</u>	0.7422	**0.7592**	0.6993
Diaphragm	0.2513	<u>0.8994</u>	0.7927	0.7751	**0.9833**	0.8863	0.8457
Goolam	0.6617	0.5379	**0.7220**	<u>0.6784</u>	0.6403	0.4194	0.5379
Lung	0.3897	0.3946	0.4913	0.1129	0.4114	<u>0.4340</u>	**0.5193**
Melanoma	0.4409	0.3541	0.4455	0.3243	<u>0.4823</u>	0.4206	**0.5165**
Pollen	0.7558	0.4473	**0.8179**	0.6062	0.4473	<u>0.6526</u>	0.6385
Romanov	0.3889	0.4982	0.2974	0.4926	**0.5167**	0.4911	<u>0.4985</u>
Trachea	0.4362	0.2182	0.4306	**0.7786**	0.2314	0.5578	<u>0.5724</u>
Wang	0.9022	0.9262	0.9122	0.9279	**0.9524**	0.9408	<u>0.9436</u>
Average	*0.4956*	*0.5247*	*0.5738*	*0.5902*	*0.5979*	***<u>0.6023</u>***	***0.6307***

Table S3. *ARI values of scZiva and five baseline methods.*
Bold and underlined values denote the best and second-best results, respectively.

NMI	AcImpute	scRMD	Raw	SAE-Impute	CL-Impute	scZiva	SAVER
Baron	0.5370	0.5910	<u>0.7342</u>	0.6707	0.6962	0.7053	**0.7610**
Bladder	0.5548	0.5651	0.6160	<u>0.8003</u>	**0.8090**	0.7335	0.7886
Diaphragm	0.3654	0.7437	0.8780	0.8073	<u>0.8621</u>	0.8092	**0.9690**
Goolam	0.6146	0.6673	0.6827	**0.8333**	0.6457	0.6827	<u>0.7965</u>
Lung	0.5683	0.6661	0.6839	0.3998	<u>0.7166</u>	**0.7437**	0.7071
Melanoma	0.6107	0.5720	0.5467	0.4941	0.5817	<u>0.6248</u>	**0.6272**
Pollen	0.8426	**0.8888**	0.7499	0.8094	<u>0.8373</u>	0.8328	0.7499
Romanov	0.4647	0.3402	<u>0.5303</u>	0.4854	0.5254	0.4884	**0.5480**
Trachea	0.5029	0.4845	0.4705	<u>0.6119</u>	0.6101	**0.6700**	0.4890
Wang	0.8255	0.8418	0.8617	0.8601	<u>0.8811</u>	0.8754	**0.9009**
Average	*0.5887*	*0.6361*	*0.6754*	*0.6772*	*0.7165*	*<u>0.7166</u>*	***0.7337***

Table S4. *NMI values of scZiva and five baseline methods.*
Bold and underlined values denote the best and second-best results, respectively.

6. Average overlap proportion over various top DEGs

Fig. S1 shows that scZiva achieves the highest average overlap proportion for 7 out of 8 cases of top DEGs.

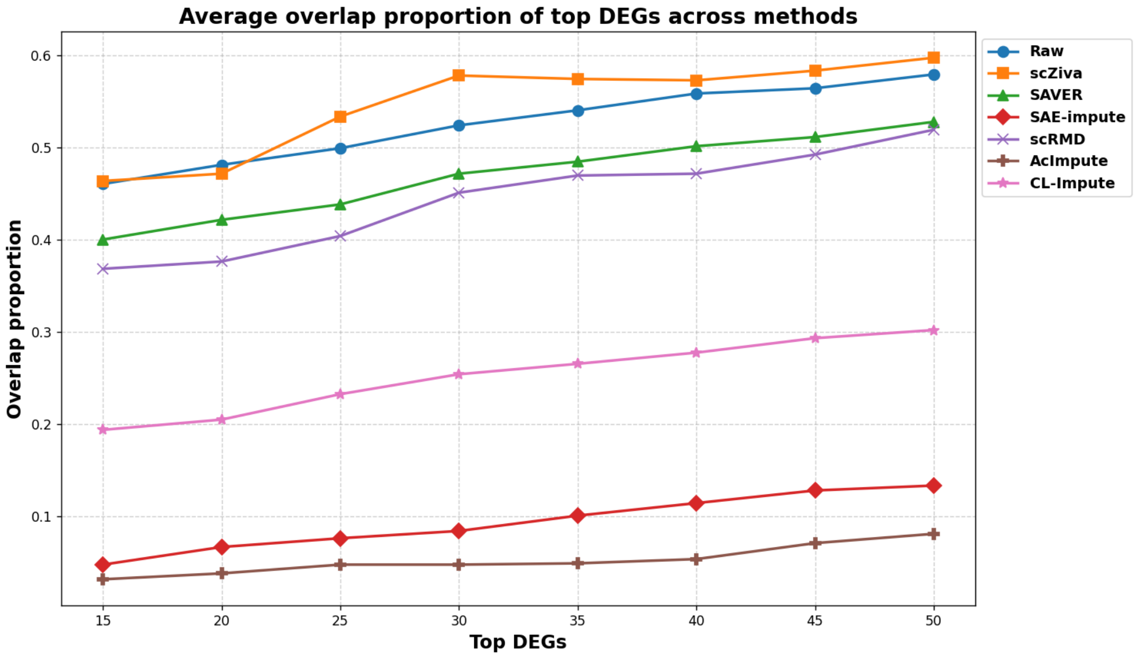

Figure S1*. Average overlap proportion over various top DEGs.*

The analysis of the top 30 differentially expressed genes (DEGs) between DEC and TB:

As presented in Figure S2, and Table S5, scZiva demonstrates a significant advantage in biological accuracy. Specifically, the number of genes overlapping with the gold-standard bulk RNA-seq data increased from 13 in the raw dataset to 20 after scZiva imputation, representing a 53% improvement. scZiva not only increases the quantity of differential expression analysis but also restored missing signals by recovering DEC markers (*CER1*, *CXCR4 [8],[9]*) which were lost to dropout noise in the raw data.

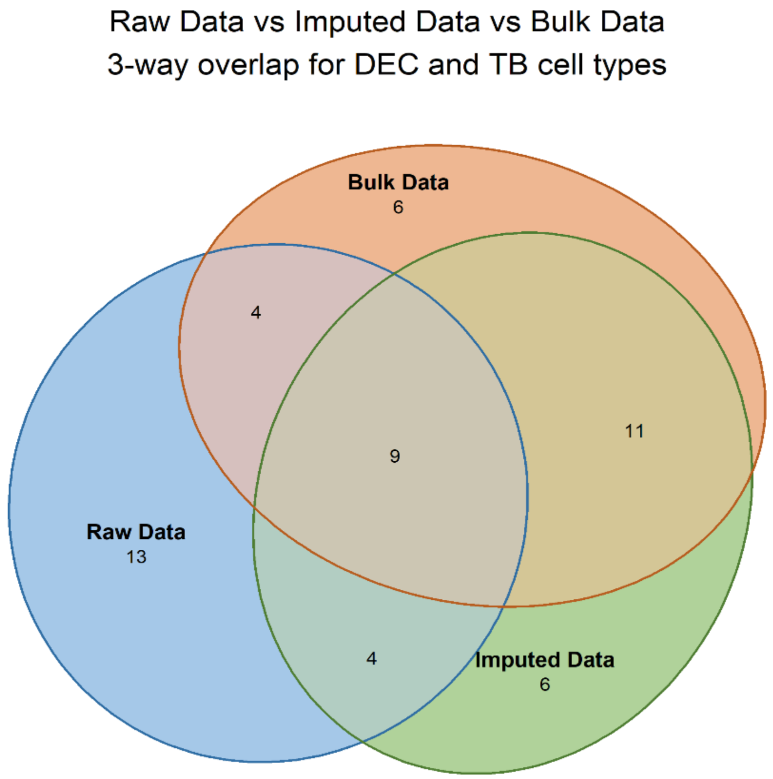

Figure S2*. Comparison of the top 30 DEGs overlapping between scRNA-seq (Raw and Imputed data by scZiva) and bulk RNA-seq for DEC and TB cell types.*

Raw data vs Bulk data	*COL12A1, PLSCR5, PAPPA2, GABRP, THRB, TRIM55, EPAS1, VGLL1, KCNMA1, MTUS2, GRHL2 LGALS3, MEIS1*
Imputed data vs Bulk data	*TRIM55, THRB, EPAS1, **VTCN1, IGFBP3**, LGALS3, VGLL1, **LRP2, CXCR4, LEFTY2**, PAPPA2, **ZIC2**, GRHL2, **PTN**, GABRP, **CER1, CYP26A1**, MEIS1, **IL34, OTX2***

Table S5*. Lists of the top 30 DEGs overlapping between scRNA-seq (Raw and Imputed data by scZiva) and bulk RNA-seq for DEC and TB cell types. The bolded gene names represent the 11 additional genes recovered correctly by scZiva compared to the case of raw data.*

7. The expression levels of housekeeping genes and marker genes

This section presents the illustration for the imputation of scZiva on known housekeeping genes (*HPRT1*, *SDHA*) and marker genes (*CXCR4*, *CD34*, *SOX2*) in GSE75748 single-cell dataset. The genes have zero expression values in several cells which are imputed by scZiva. Noted that *CXCR4*, *CD34* genes are marker of DEC and

EC cell types, respectively, while *SOX2* gene is a transcription factor and also a marker gene of NPC, H1 and H9, reported in original study of the dataset [8] and CellMarker 2.0 [9].

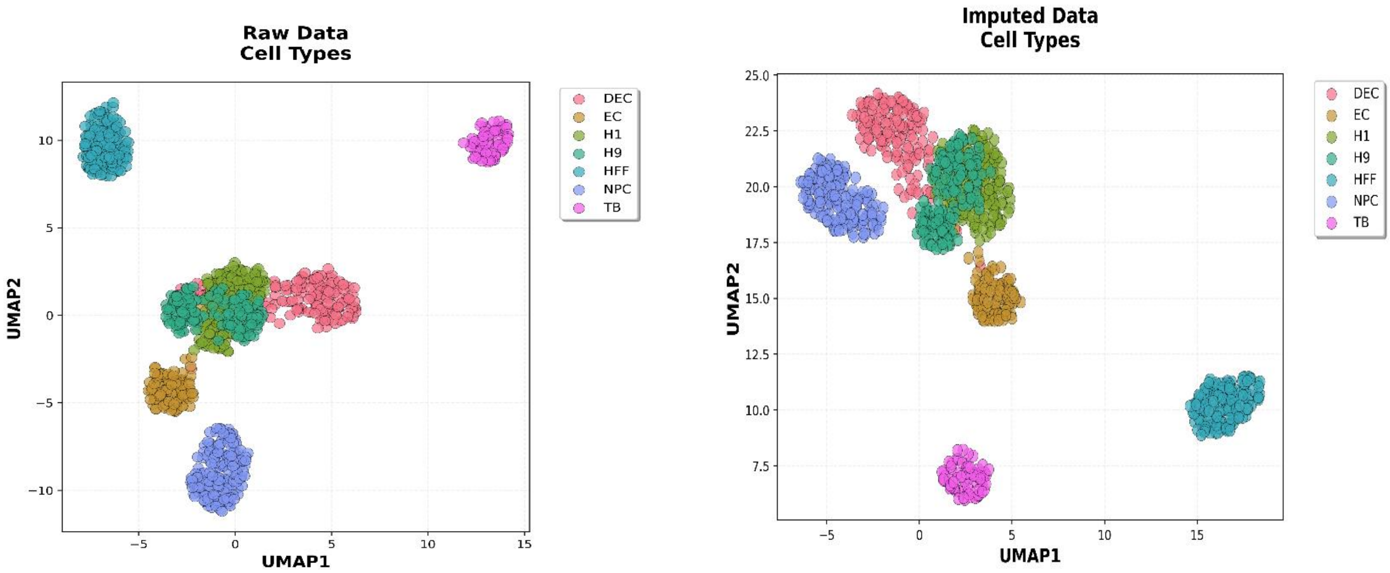

a) b)

Figure S3. *UMAP visualization of cells colored by ground truth cell types for (a) Raw data and (b) imputed data.*

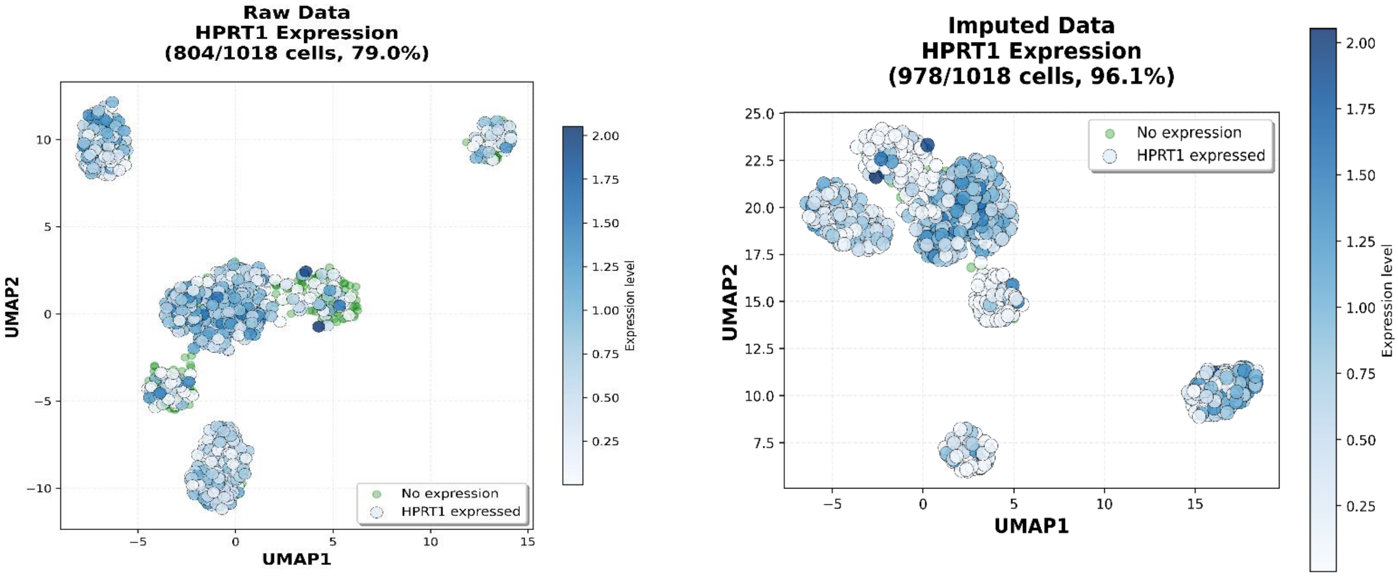

a) b)

Figure S4. *UMAP visualization of cells colored by HPRT1 gene expression levels for (a) raw data and (b) imputed data.*

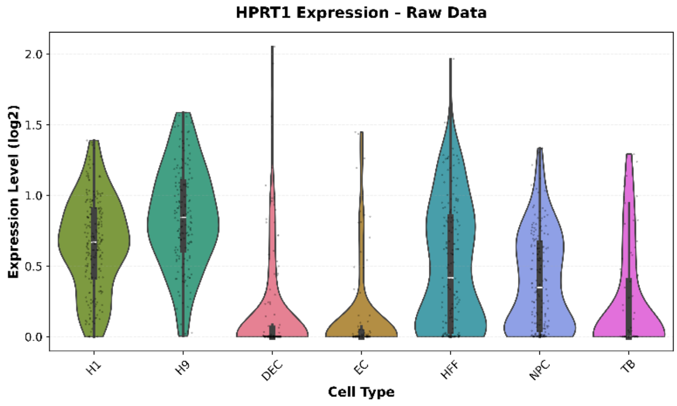

a)

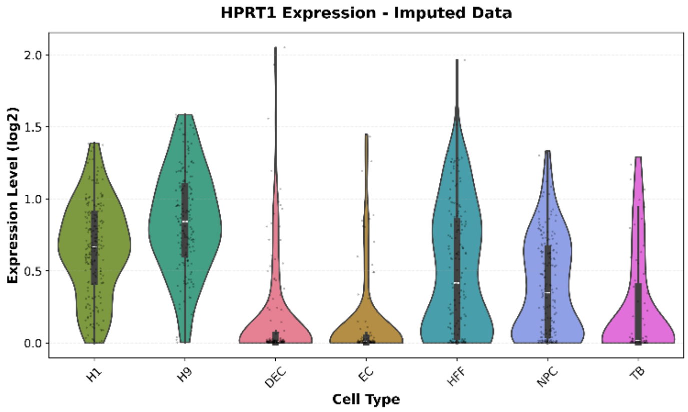

b)

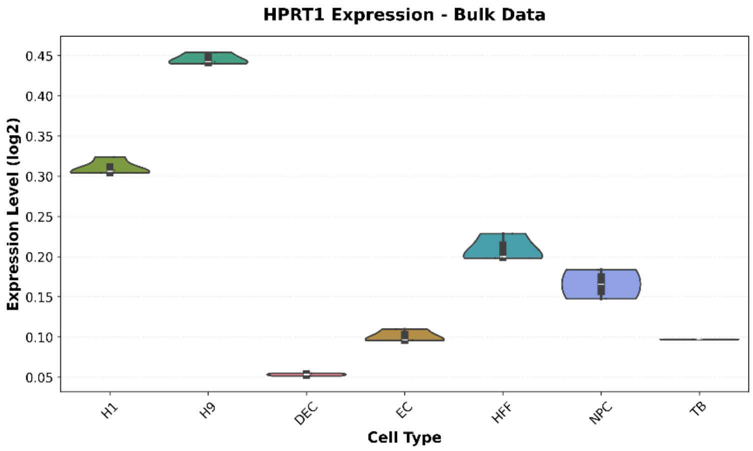

c)

Figure S5. *Violin plots of HPRT1 gene expression across cell types for (a) raw data, (b) imputed data, and (c) bulk data.*

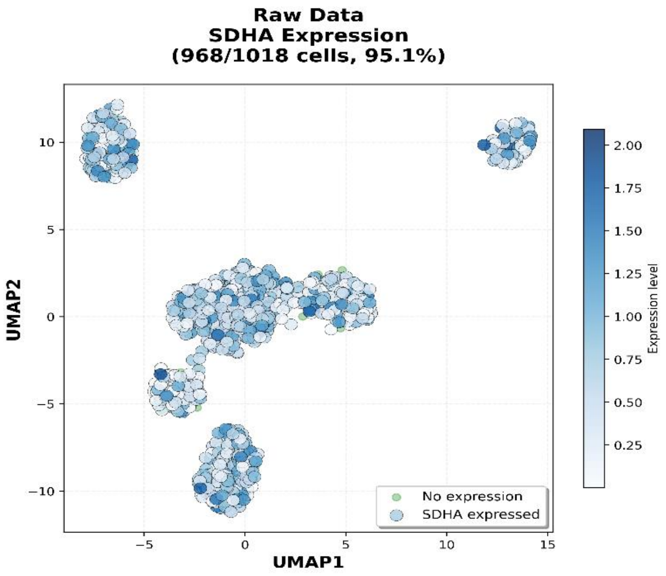

a)

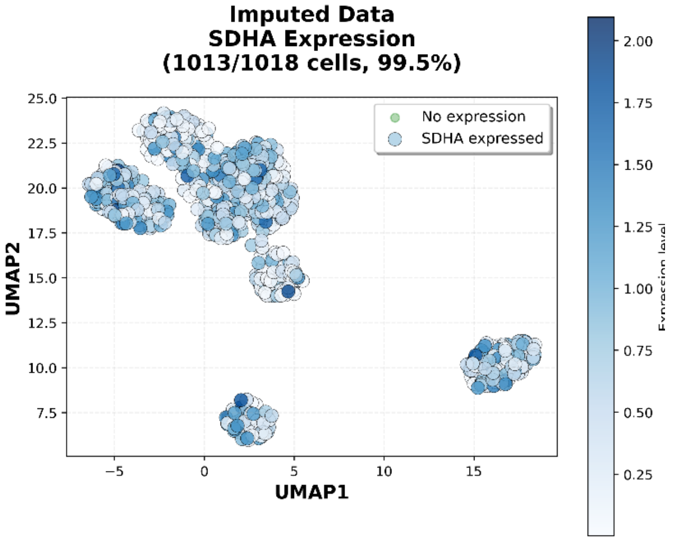

b)

Figure S6. *Umap visualization of cells colored by SDHA gene expression levels for (a) raw data and (b) imputed data.*

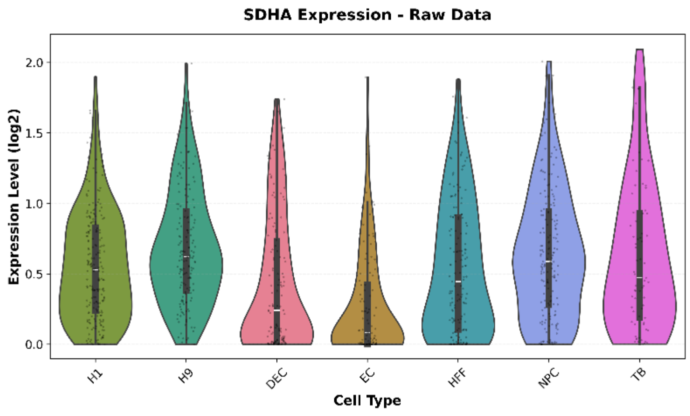

a)

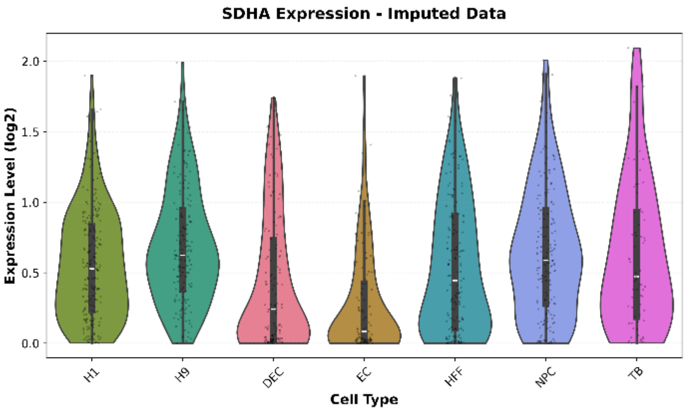

b)

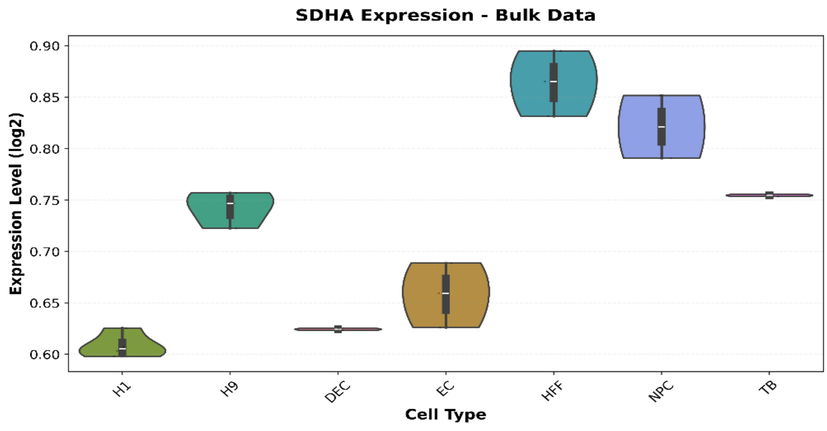

c)

Figure S7. *Violin plots of SDHA gene expression across cell types for (a) raw data, (b) imputed data, and (c) bulk data.*

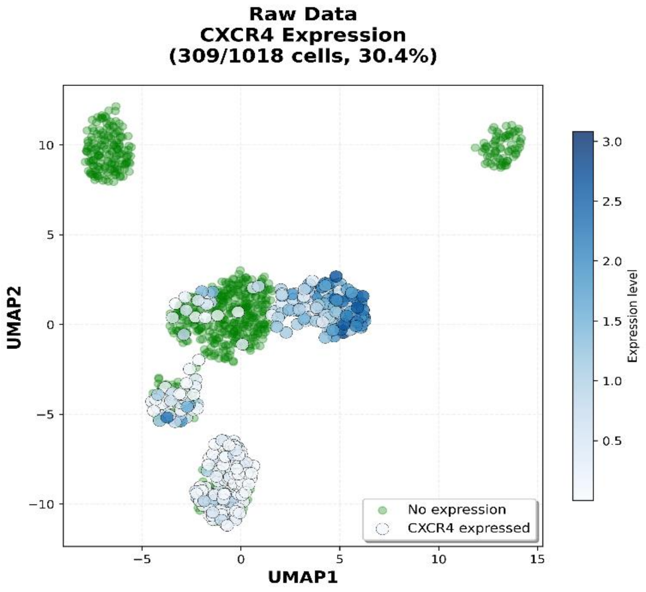

a)

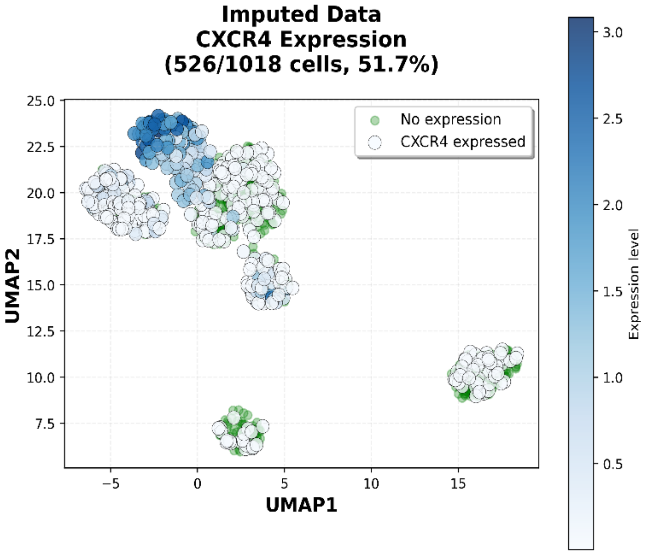

b)

Figure S8. *Umap visualization of cells colored by CXCR4 gene expression levels for (a) raw data and (b) imputed data.*

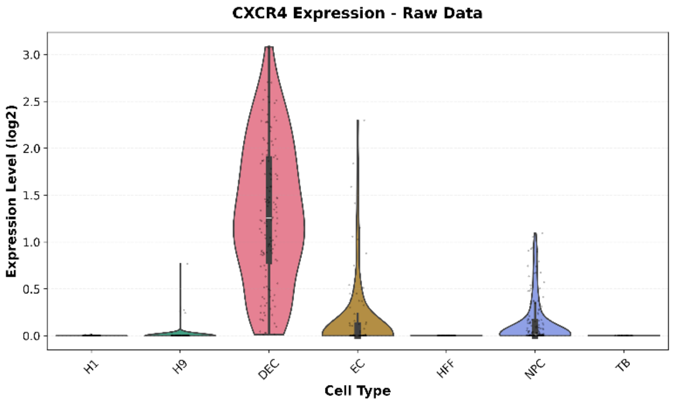

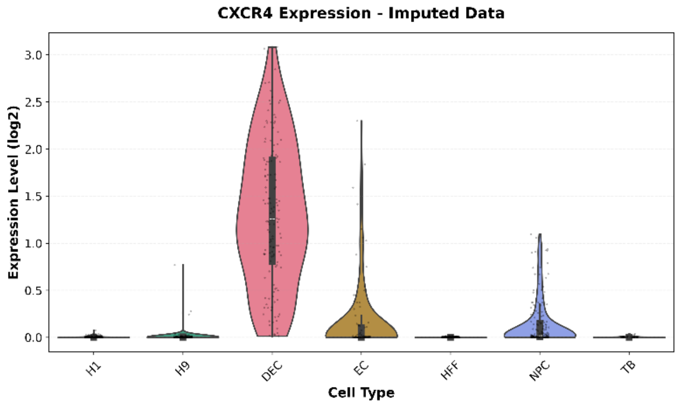

a) b)

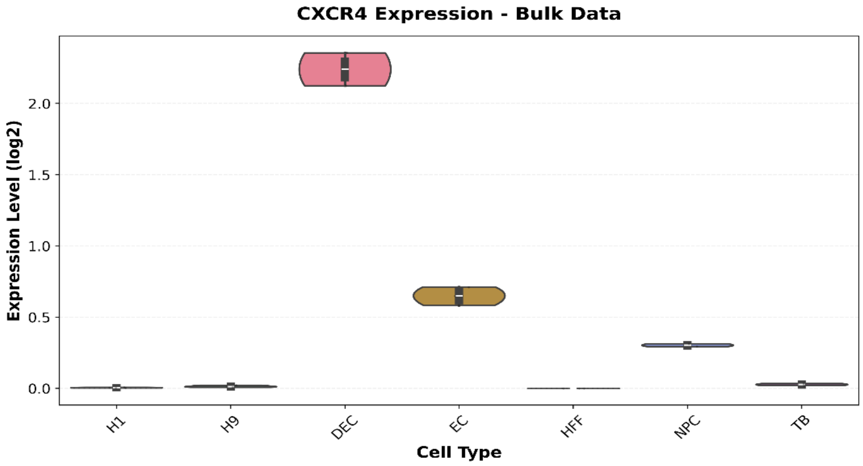

Figure S9. *Violin plots of CXCR4 gene expression across cell types for (a) raw data, (b) imputed data, (c) bulk data.*

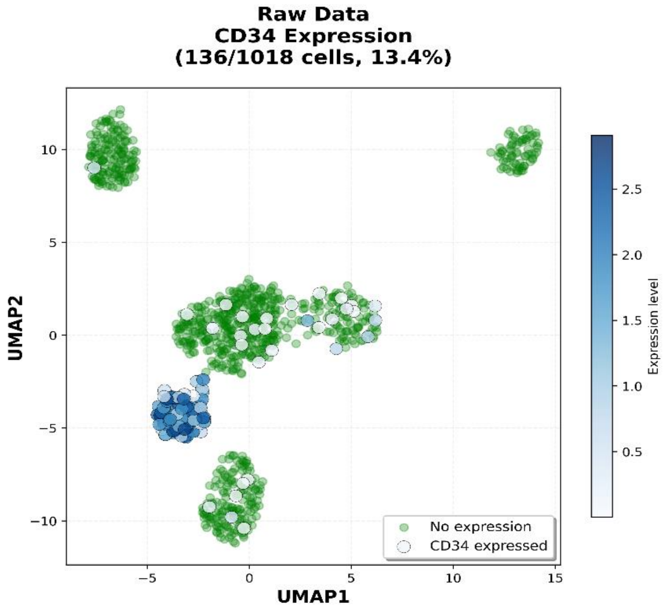

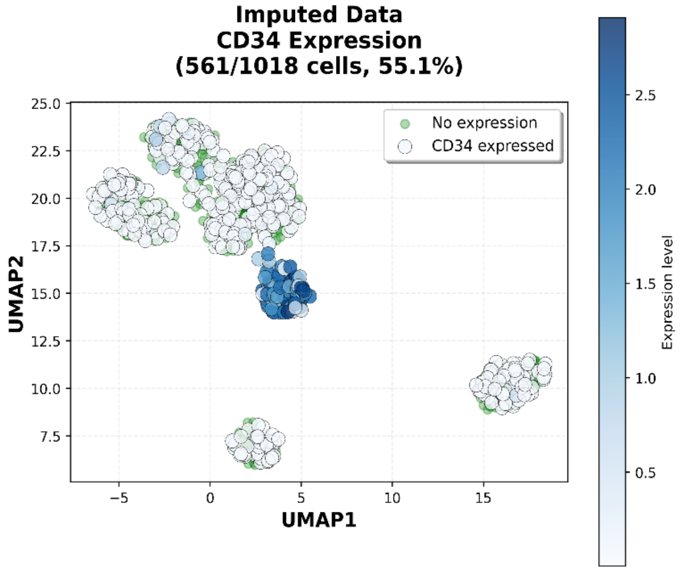

a) b)

Figure S10. *Umap visualization of cells colored by CD34 gene expression levels for (a) raw data and (b) imputed data.*

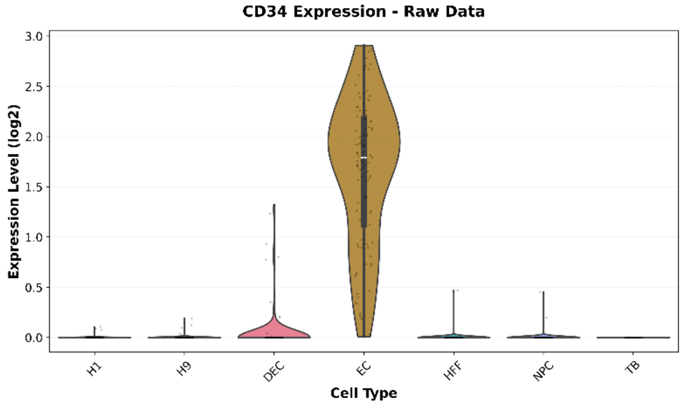

a)

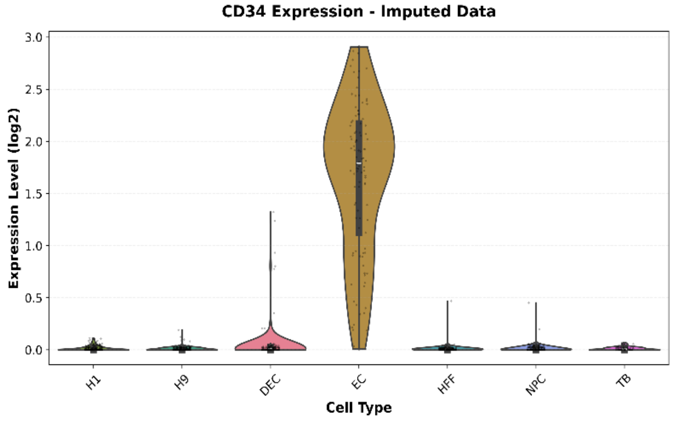

b)

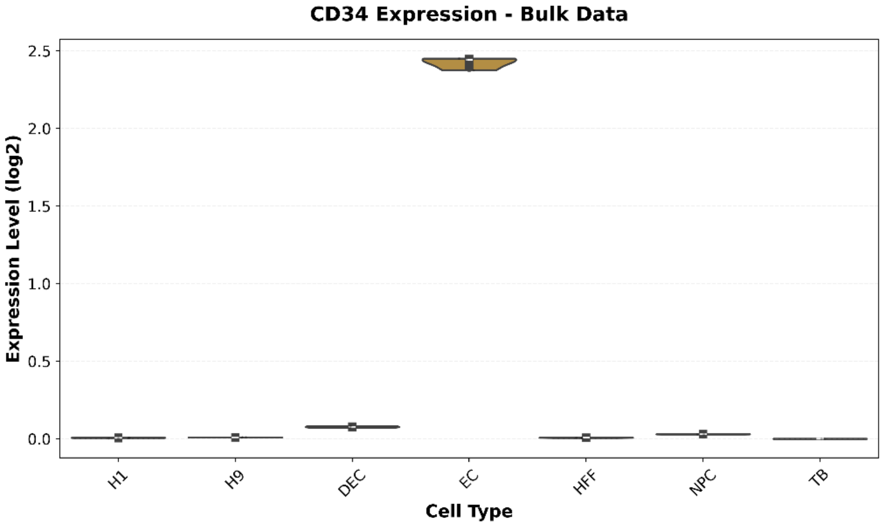

Figure S11. *Violin plots of CD34 gene expression across cell types for (a) raw data, (b) imputed data, and (c) bulk data.*

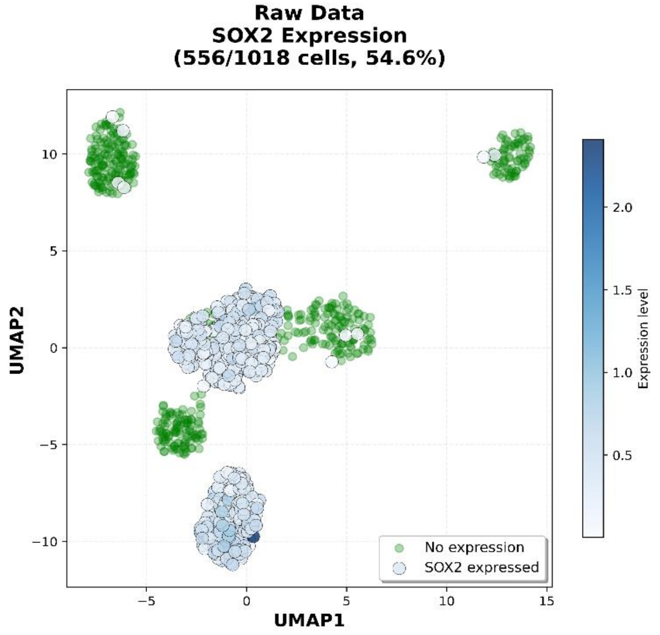

a)

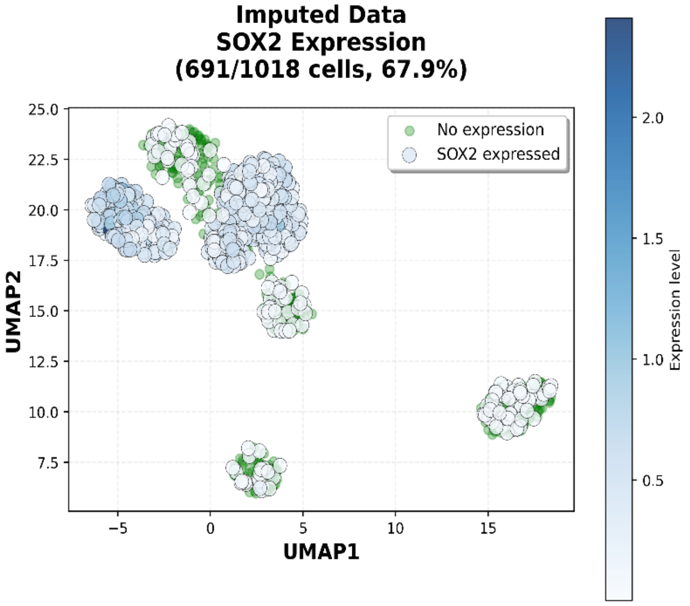

b)

Figure S12. *Umap visualization of cells colored by SOX2 gene expression levels for (a) raw data and (b) imputed data.*

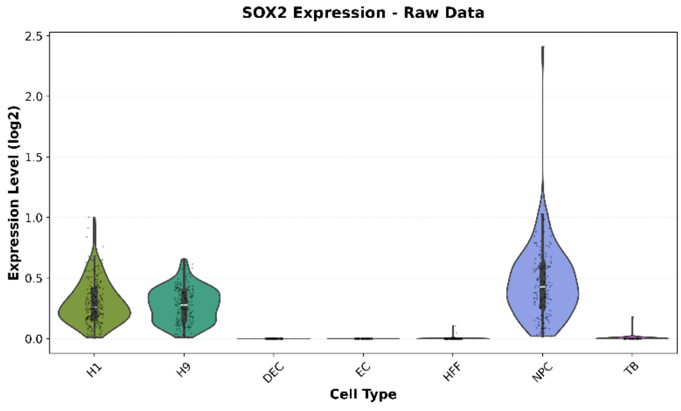

a)

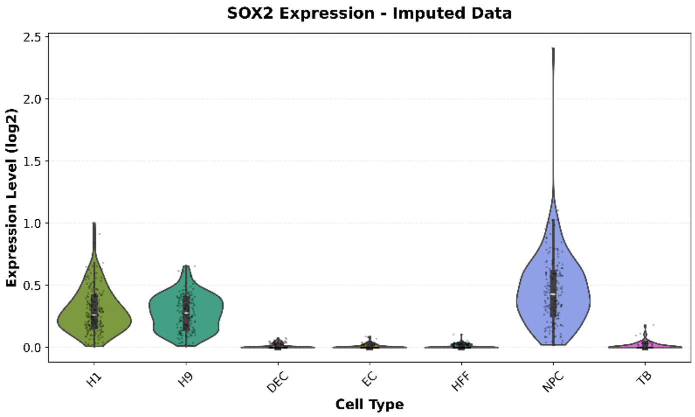

b)

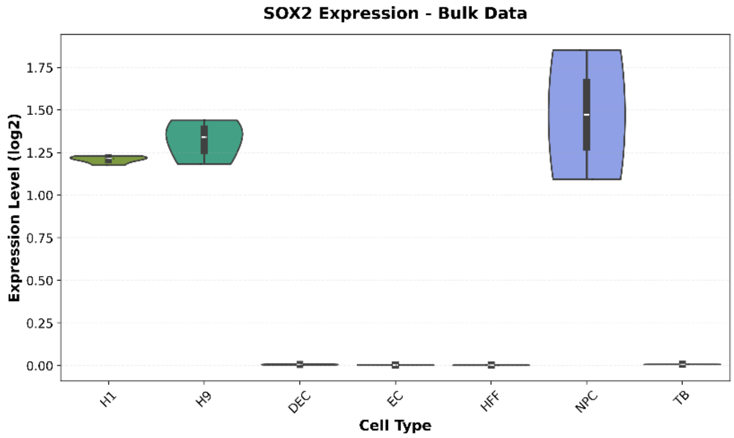

Figure S13. *Violin plots of SOX2 gene expression across cell types for (a) raw data, (b) imputed data, and (c) bulk data.*

8. Cell trajectory inference of CL-Impute, SAE-Impute and AcImpute

CL-Impute

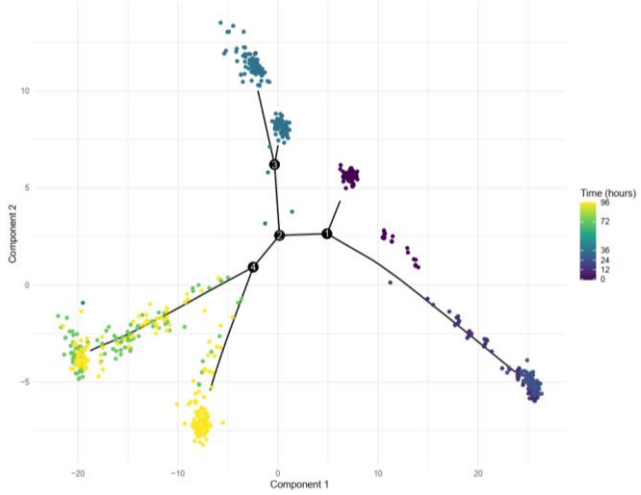

a)

SAE-Impute

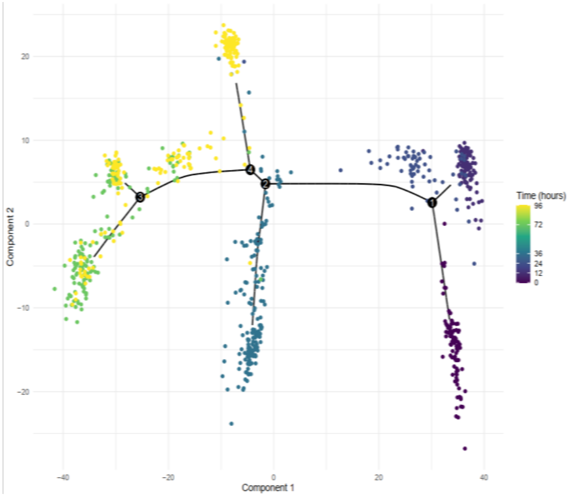

b)

AcImpute

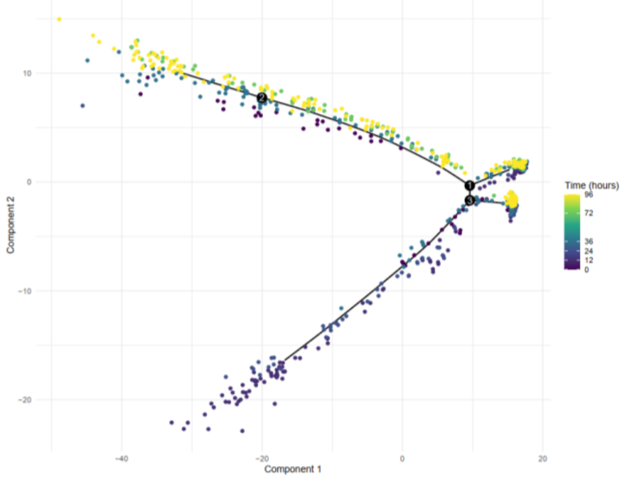

c)

Figure S14. *The visualization of trajectory by time with the support of imputation methods: (a)CL-Impute, (b) SAE-Impute, and (c) AcImpute.*

9. Computational performance

Table S5 presents running time, RAM, and GPU memory required by scZiva and baseline methods on GSE75748 single-cell dataset. The experiments were conducted on a system with 128 GB RAM, Intel Core i9 CPUs, and an NVIDIA Quadro RTX 400 GPU (8 GB memory). It can be seen that scRMD is the fastest algorithm, while AcImpute, and SAVER need the highest running time. Except for SAVER, memory usages of the remaining methods are similar. scZiva achieves the lowest running time while requiring comparable GPU memory usage among deep learning methods (CL-Impute, SAE-Impute, and scZiva). Runing time of scZiva is 77 seconds in which reordering time cost is 23 seconds.

**Methods**	**Running time (seconds)**	**RAM**	**GPU memory**
**scRMD**	37	6.4 GB	x
**AcImpute**	2834	6.7 GB	x
**SAVER**	1780	9 GB	x
**CL-Impute**	346	7 GB	2.2 GB
**SAE-Impute**	976	6 GB	0.8 GB
**scZiva**	77	6 GB	0.8 GB

Table S6. *Computational performance of scZiva and baseline methods.*

10. Sensitivity analysis of the threshold τ

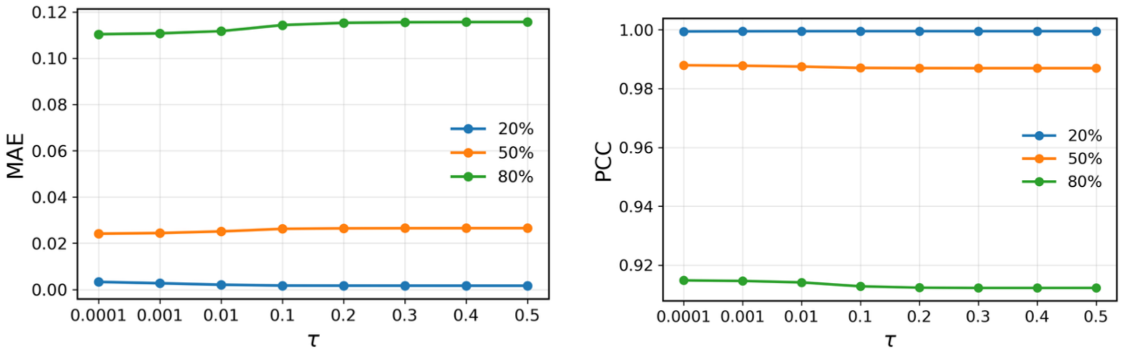

Figure S15. *Sensitivity analysis of the selective imputation threshold τ. MAE (left) and PCC (right) are evaluated across different τ values under simulated dropout rates of 20%, 50%, and 80%.*

To evaluate the robustness of scZiva with respect to the selective imputation threshold τ, we conducted a sensitivity analysis over a broad range of values τ. The analysis was performed on Tung datasets with various dropout rates. Performance was assessed using MAE and PCC. As shown in Figure S15, scZiva exhibits stable performance across a wide interval of τ.

For all dropout settings, MAE varies only marginally when τ ranges between 0.01 and 0.1. Similarly, PCC remains nearly constant across the tested values, indicating that the reconstruction quality is not overly sensitive to the exact threshold choice.

These results suggest that while minor improvements may be achieved through fine-tuning τ, the method remains robust within a reasonable range. In practice, we recommend selecting τ within the interval [0.0001, 0.01], where performance remains consistently stable across different dropout conditions.

References

[1] Baron M, Veres A, Wolock SL, Faust AL et al. A Single-Cell Transcriptomic Map of the Human and Mouse Pancreas Reveals Inter- and Intra-cell Population Structure. *Cell Syst* 2016 Oct 26;3(4):346-360.e4. PMID: 27667365

[2] Schaum, N., Karkanias, J., Neff, N. F., May, A. P., Quake, S. R., Wyss-Coray, T, van Weele, L. J. *et al* (2018). Single-cell transcriptomics of 20 mouse organs creates a Tabula Muris: The Tabula Muris Consortium. *Nature*, *562*(7727), 367.

[3] Goolam, M., Scialdone, A., Graham, S.J., Macaulay, I.C., Jedrusik, A., Hupalowska, A., Voet, T., Marioni, J.C. and Zernicka-Goetz, M., 2016. Heterogeneity in Oct4 and Sox2 targets biases cell fate in 4-cell mouse embryos. Cell, 165(1), pp.61-74.

[4] Tirosh, I., Izar, B., Prakadan, S. M., Wadsworth, M. H., Treacy, D., Trombetta, J. J., Garraway, L. A. *et al* (2016). Dissecting the multicellular ecosystem of metastatic melanoma by single-cell RNA-seq. *Science*, *352*(6282), 189-196.

[5] Pollen, A. A., Nowakowski, T. J., Shuga, J., Wang, X., Leyrat, A. A., Lui, J. H., West, J. A. *et al* (2014). Low-coverage single-cell mRNA sequencing reveals cellular heterogeneity and activated signaling pathways in developing cerebral cortex. *Nature biotechnology*, *32*(10), 1053-1058.

[6] Romanov, R.A., Zeisel, A., Bakker, J., Girach, F., Hellysaz, A., Tomer, R., Alpar, A., Mulder, J., Clotman, F., Keimpema, E. and Hsueh, B., 2017. Molecular interrogation of hypothalamic organization reveals distinct dopamine neuronal subtypes. *Nature neuroscience*, 20(2), pp.176-188.

[7] Wang, Y., Tang, Z., Huang, H., Li, J., Wang, Z., Yu, Y., Zhang, C., Li, J., Dai, H., Wang, F. and Cai, T., 2018. Pulmonary alveolar type I cell population consists of two distinct subtypes that differ in cell fate. *Proceedings of the National Academy of Sciences*, 115(10), pp.2407-2412.

[8] Chu, L.F., Leng, N., Zhang, J., Hou, Z., Mamott, D., Vereide, D.T., Choi, J., Kendziorski, C., Stewart, R. and Thomson, J.A., 2016. Single-cell RNA-seq reveals novel regulators of human embryonic stem cell differentiation to definitive endoderm. *Genome biology*, 17(1), p.173.

[9] Congxue Hu, Tengyue Li, Yingqi Xu, Xinxin Zhang, Feng Li, Jing Bai, Jing Chen, Wenqi Jiang, Kaiyue Yang, Qi Ou, et al. Cellmarker 2.0: an updated database of manually curated cell markers in human/mouse and web tools based on scrna-seq data. *Nucleic acids research*, 51(D1):D870 - D876, 2023
